# Alterations in peripheral blood B cell subsets and their clinical significance in systemic lupus erythematosus

**DOI:** 10.1038/s41598-026-37415-z

**Published:** 2026-01-27

**Authors:** Jingyuan Huang, Ziling Xu, Xiaodan Zhang, Xin Wang, Liangjun Zhang

**Affiliations:** Department of Laboratory Medicine, Zigong First People’s Hospital, Zigong, Sichuan China

**Keywords:** Systemic lupus erythematosus, B cell subsets, Memory B cells, Plasmablasts, Diagnostic biomarker, Biomarkers, Diseases, Immunology, Rheumatology

## Abstract

Systemic lupus erythematosus (SLE) is characterized by B-cell dysregulation and autoantibody production. This study aimed to characterize the alterations in peripheral blood B-cell subsets across different states of SLE and to evaluate their diagnostic value and association with specific autoantibodies.A total of 84 participants were enrolled, comprising 64 SLE patients (including 20 newly diagnosed, 27 stable, and 17 with lupus nephritis) and 20 healthy controls (HCs). Peripheral blood B-cell subsets, including naïve B-cells, memory B-cells, and plasmablasts, were analyzed by flow cytometry. Their diagnostic performance was assessed using Receiver Operating Characteristic (ROC) curve analysis. Associations with anti-nuclear antibody (ANA), anti-dsDNA, and anti-Sm antibody status were also evaluated.Compared to HCs, SLE patients exhibited significant disturbances in B-cell homeostasis. The most consistent finding was a profound decrease in the absolute frequency of memory B cells across all patient groups (newly diagnosed, stable, and LN; all p < 0.001). Newly diagnosed patients showed a significant expansion of plasmablasts (p = 0.017), which was less pronounced in stable and LN groups. ROC analysis demonstrated that the absolute memory B-cell frequency (memory B%) had outstanding diagnostic performance for SLE (AUC = 0.905, 80% sensitivity, 80% specificity). Furthermore, higher anti-nuclear antibody (ANA) titers and anti-dsDNA positivity were significantly associated with a decreased absolute naïve B-cell count and an increased relative proportion of plasmablasts. Anti-Sm positivity was specifically linked to a higher plasmablast proportion (p = 0.006).Our findings highlight a marked disruption of peripheral B-cell subsets in SLE, particularly a persistent reduction in memory B cells and an expansion of plasmablasts in active disease. Memory B cells serve as an excellent diagnostic biomarker, while plasmablast expansion is closely associated with specific autoantibodies, underscoring the pivotal role of aberrant B-cell differentiation in SLE immunopathogenesis.

## Introduction

Systemic lupus erythematosus (SLE) is a complex autoimmune disorder that poses a serious threat to human health. Studies have reported that the mean age of SLE patients is 43.03 years, with females accounting for 81.33% of cases^1^. SLE manifests with diverse clinical presentations and can affect multiple organ systems, including the skin, joints, kidneys, and nervous system. Diagnosis is typically based on clinical features and laboratory tests, such as anti-nuclear antibody (ANA) testing^2^. Early diagnosis and timely intervention are crucial for improving patient outcomes.

In autoimmune diseases, B cells play a central role in coordinating antigen presentation, cytokine production, and autoantibody secretion—the latter achieved through their differentiation into antibody—producing plasmablasts and plasma cells^3^. Growing evidence suggests that the pathogenesis of SLE is closely linked to dysregulation in B-cell development. It is hypothesized that exposure to foreign antigens, possibly through molecular mimicry with self-antigens, activates B cells, leading to their proliferation and differentiation into plasma cells. These cells then produce large quantities of autoantibodies, resulting in inflammatory damage across various tissues and organs^4^. This implies that peripheral B cells in SLE patients may exhibit altered differentiation states, and such aberrant B-cell dynamics may play an integral role in disease initiation and progression^4^.

Therefore, a deeper investigation into the distribution of B-cell subsets in autoimmune conditions and their specific roles in SLE is of great importance. This study aims to analyze changes in naïve B cells, memory B cells, and plasmabasts during the course of SLE and to explore their correlations with clinical features. Through this approach, we seek to elucidate the significance of different B-lymphocyte subpopulations in the development and progression of SLE.

## Patients and methods

### Patients

A total of 64 patients diagnosed with systemic lupus erythematosus (SLE) at Zigong First People’s Hospital between January 2023 and December 2024 were enrolled in this study. The study was performed in accordance with relevant guidelines and regulations and was approved by the Ethics Committee of The First People’s Hospital of Zigong (NO.03202024), and written informed consent was obtained from all participants. These patients were categorized into three mutually exclusive groups based on their dominant clinical status at enrollment: newly diagnosed SLE without major organ involvement (n = 20), stable SLE (n = 27), and active lupus nephritis (LN) (n = 17). Group definitions were as follows: 1) Newly diagnosed SLE: patients meeting the 2019 EULAR/ACR classification criteria^5^ for the first time, who had not received any prior immunosuppressive therapy or glucocorticoids (dosage > 10 mg/day prednisone equivalent) for SLE, and without clinical or laboratory evidence of active nephritis, neuropsychiatric involvement, or severe hematological abnormalities. 2) Stable SLE: patients with a confirmed SLE diagnosis for at least 6 months, on stable treatment for a minimum of 3 months, and with a clinical SLE Disease Activity Index (SLEDAI) score ≤ 4. 3) Active LN group: patients with a clinically active renal syndrome (e.g., proteinuria ≥ 0.5 g/24 h and/or active urinary sediment) and a renal biopsy performed within the past 6 months confirming LN according to the 2003 ISN/RPS classification^6^. Any newly diagnosed patient who also met the criteria for active LN was assigned to the LN group. Additionally, 20 healthy individuals undergoing routine health examinations were recruited as the control group. The inclusion criterion for the patient groups was conformity with the 2019 European Alliance of Associations for Rheumatology/American College of Rheumatology (EULAR/ACR) classification criteria for SLE^5^. The diagnosis of LN was confirmed by renal biopsy, and the pathological classification was based on the 2003 International Society of Nephrology/Renal Pathology Society (ISN/RPS) revised criteria for LN^6^. Exclusion criteria applied to all participants included: pregnancy or lactation; coexistence of other autoimmune diseases (e.g., rheumatoid arthritis, Sjögren’s syndrome, systemic sclerosis); severe comorbidities affecting major organ systems (including significant cardiovascular, hepatic, or pulmonary diseases); active malignancy; history of severe infection within one month prior to enrollment or requiring intravenous antibiotic therapy; and presence of renal diseases attributable to other causes (such as diabetic nephropathy, hypertensive nephropathy, or primary glomerulonephritis).

### Methods

**Fig. 1:**
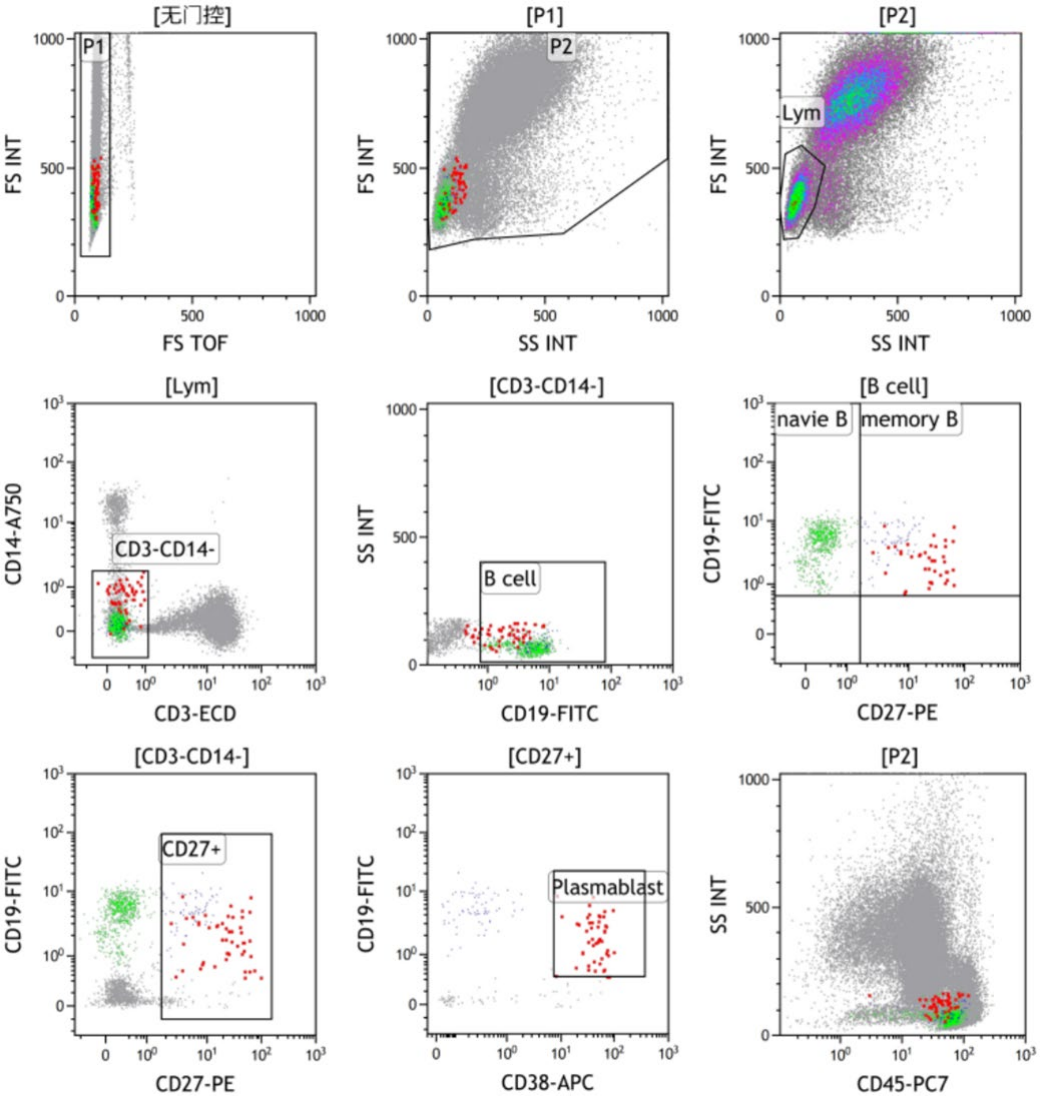
Gating strategy for B lymphocyte subsets in peripheral blood samples

EDTA-K2 anticoagulated peripheral blood samples were collected from all participants for flow cytometric analysis. Briefly, 100 μL of whole blood was aliquoted into a flow tube and stained with 5 μL each of the following fluorescently labeled antibodies: CD19-FITC, CD27-PE, CD3-ECD, CD38-APC, CD45-PC7, and CD14-APC750 (all from Beckman Coulter, California, USA). The mixture was thoroughly vortexed and incubated for 20 min at room temperature, protected from light. Following incubation, 500 μL of lysing solution (Beckman Coulter, California, USA) was added to the tube. After gentle mixing, the samples were lysed for 10 min in the dark. Phosphate-buffered saline (PBS, Beckman Coulter, California, USA) was then added to stop the reaction, and the cells were centrifuged at 500 × g for 5 min. The supernatant was carefully discarded, and the cell pellet was washed once and resuspended in PBS for acquisition. Data acquisition was performed using a Navios flow cytometer (Beckman Coulter, 8 colors/2 lasers). The resulting data were analyzed with Kaluza analysis software. Initial data acquisition involved the elimination of cell doublets by gating on single cells using a plot of FS TOF versus FS INT. Subsequently, a primary gate was set on the scatter plot of FSC versus SSC to identify the population of nucleated cells while excluding cellular debris. The lymphocytes population was gated based on FSC and SSC characteristics. From this parent gate, a subpopulation of CD14^-^ CD3^-^ cells was selected to exclude monocytes and T lymphocytes. B cell subsets were identified from this lineage-negative population as three mutually exclusive populations: naïve B cells (CD19^+^ CD27^--^), memory B cells (CD19^+^ CD27^+^ CD38^-^/low), and plasmablasts (CD19^+^ CD27^+^ CD38high). A schematic diagram of the gating strategy is presented in Fig. 1.

Antinuclear antibodies (ANA) were screened by indirect immunofluorescence (IFA) using kits from EUROIMMUN (Lübeck, Germany). An ANA titer of ≥ 1:100 was defined as positive, while a titer of < 1:100 was considered negative. Specific antibodies, including anti-double-stranded DNA (anti-dsDNA) and anti-Smith (anti-Sm), were detected using a standardized immunoblot (line blot) assay for extractable nuclear antigens (ENA) from EUROIMMUN (Lübeck, Germany). Positivity was determined according to the presence of specific, manufacturer-defined bands on the immunoblot strip.

### Statistical analyses

We used SPSS (version 25.0; IBM Corp.) and GraphPad Prism 5.01 software was used to further analyze and visualize the summarized data. Non-normal distribution was observed for all cell types, so the median and interquartile was used to describe the data for each group. For non-normally distributed lymphocyte subsets, comparisons between different two groups were performed using Mann–Whitney tests. The Kruskal–Wallis test was used for comparisons between multiple groups. Pearson’s correlation test was used for the correlation analysis. Receiver operating characteristic (ROC) curves were analyzed to determine the diagnostic value of the disease. Statistical significance was set at P < 0.05.

## Results

### Comparison of clinical and demographic parameters

The demographic, clinical, and therapeutic characteristics of all study participants are summarized in Table 1. There were no statistically significant differences in age or sex distribution among the SLE patient groups and healthy controls (HCs). As per the study design, significant differences were observed in key disease parameters across the SLE subgroups. Disease duration was shortest in the newly diagnosed group (median: 0 month) and longest in the stable SLE group (median: 48 months). Disease activity, as measured by the SLEDAI-2 K score, was highest in the LN SLE group (median: 16), followed by the Newly diagnosed group (median: 8), and was consistently low in the Stable SLE group (median: 2; P < 0.001). While the proportions of patients positive for anti-dsDNA antibodies or with hypocomplementemia did not differ significantly between SLE groups, the presence of clinically significant proteinuria (≥ 1 +) was exclusively observed in the LN SLE group (100%, P < 0.001). Renal biopsy for all LN patients confirmed ISN/RPS Class III (n = 4), Class IV (n = 9), Class V (n = 3), or mixed Class III + V (n = 1) pathology. Treatment patterns aligned with the groups’ clinical definitions. No patient in the Newly diagnosed SLE group was on glucocorticoids or immunosuppressants at enrollment. In contrast, the majority of patients in the Stable SLE and LN SLE groups were receiving these treatments (glucocorticoids: 88.9% and 94.1%; immunosuppressants: 96.3% and 88.2%, respectively; P < 0.001 for both comparisons). This profile confirms the successful stratification of patients into distinct cohorts representing different phases and severities of SLE.

**Table 1:** Demographic and clinical characteristics of the study cohorts.

Characteristics	HCs (n = 20)	Newly diagnosed SLE (n = 20)	Stable SLE (n = 27)	LN SLE (n = 17)	P value
Sex, n (%)					0.382
Male	4 (20)	6 (30)	2(7)	2 (12)	
Female	16 (80)	14 (70)	25 (93)	15 (88)	
Age, mean ± SD	46.8 ± 13.49	42.7 ± 14.06	43.11 ± 14.1	47.35 ± 9.31	0.611
Clinical parameters					
Disease duration (months), median(Q1,Q3)	-	0 (0, 1)	48 (24, 96)	18 (6, 48)	< 0.001
SLEDAI-2 K score, median(Q1,Q3)	-	8 (6, 10)	2 (0, 4)	16(12, 20)	< 0.001
Anti-dsDNA positive, n (%)	-	15 (75.0)	16 (59.3)	14 (82.4)	0.245
Hypocomplementemia, n (%)	-	17 (85.0)	18 (66.7)	15 (88.2)	0.178
Proteinuria (≥ 1 +), n (%)	-	0 (0)	0 (0)	17 (100)	< 0.001
ISN/RPS Class, n	-	-	-	Class III: 4 Class IV: 9 Class V: 3 Class III + V: 1	-
Glucocorticoids use, n (%)	-	0 (.0)	24 (88.9)	16 (94.1)	< 0.001
Immunosuppressant use, n (%)	-	0 (0)	26 (96.3)	15 (88.2)	< 0.001

### Comparison of B-cell subsets among SLE disease states and healthy controls

**Fig. 2:**
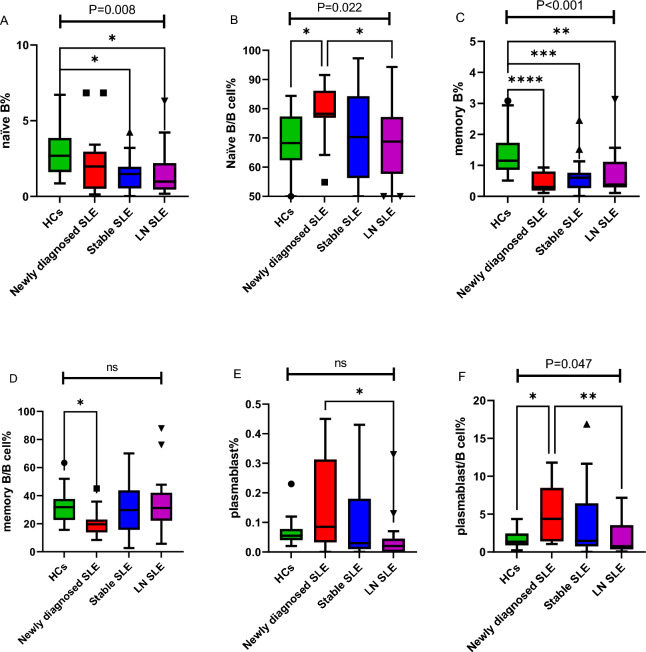
Lymphocyte subsets from healthy controls and patients with SLE across different disease stages. (**A**). Percentage of naïve B among total nucleated cells across the four groups. (**B**) Percentage of naïve B among B cells across the four groups. (**C**) Percentage of memory B among total nucleated cells across the four groups. (**D**) Percentage of memory B among B cells across the four groups. (**E**) Percentage of plasmablast among total nucleated cells across the four groups. F. Percentage of plasmablast among B cells across the four groups. Legend: Data are shown as box (IQR) and whiskers (range) plots with individual points. The horizontal line inside the box marks the median. The asterisk(s) above a bracket indicate a significant pairwise comparison between the groups connected by that bracket: *P < 0.05, **P < 0.01, ***P < 0.001, ****P < 0.0001

Analysis of peripheral blood B cell subsets revealed significant differences between patients with SLE and HCs, as well as across different SLE disease stages (see Table 2 and Fig. 2 for a summary). The pooled cohort of all SLE patients (n = 64) exhibited significant disturbances in specific B-cell populations compared to HCs (n = 20). Both the proportion of naïve B cells among total nucleated cells (naïve B%) and the frequency of memory B cells (memory B%) were markedly lower in SLE patients (median [IQR]: 1.28% [0.58–2.19] and 0.44% [0.27–0.76], respectively) than in HCs (2.69% [1.65–3.84] and 1.15% [0.86–1.58]; *p* = 0.001 and *p* < 0.001, respectively). In contrast, no significant differences were found between all SLE patients and HCs in the relative proportions of naïve or memory B cells within the total B-cell compartment, the frequency of plasmablasts, or the proportion of plasmablasts among B cells.

**Table 2:** Frequencies of B-cell subsets: comparison of all SLE patients vs. healthy controls and across SLE disease states.

Variables	HCs (n = 20)	All SLE patients (n = 64)	P value (SLE vs HC)	Newly diagnosed SLE (n = 20)	Stable SLE (n = 27)	LN SLE (n = 17)	Overall P value	Pairwise P values (post-hoc/exploratory)
Sex, n (%)			0.733				0.382	-
Male	4 (20)	10 (16)		6 (30)	2(7)	2 (12)		
Female	16 (80)	54 (84)		14 (70)	25 (93)	15 (88)		
Age, mean ± SD	46.8 ± 13.49	43.91 ± 13.10	0.372	42.7 ± 14.06	43.11 ± 14.1	47.35 ± 9.31	0.611	-
Naïve B%, median (Q1,Q3)	2.69 (1.65, 3.84)	1.28 (0.58, 2.19)	***0.001***	1.98 (0.68, 2.89)	1.48 (0.58, 1.91)	0.98 (0.49, 2.19)	***0.008***	S vs HC: 0.027, LN vs HC: 0.013, Other comparisons: NS
Naïve B/B cell%, median (Q1,Q3)	68.25 (62.8, 76.89)	72.36 (62.10, 81.45)	0.351	78.28 (77.05, 84.82)	70.31 (59.48, 84.09)	68.76 (62.1, 75.59)	***0.022***	N vs HC: 0.049, N vs LN: 0.047,Other comparisons: NS
Memory B%, median (Q1,Q3)	1.15 (0.86, 1.58)	0.44 (0.27, 0.76)	** < *0.001***	0.3 (0.21, 0.76)	0.6 (0.28, 0.74)	0.38 (0.32, 1.09)	** < *0.001***	N vs HC: < 0.001, S vs HC: < 0.001, LN vs HC: 0.008, Other comparisons: NS
Memory B/B cell%, median (Q1,Q3)	31.75 (23.11, 37.2)	25.73 (16.90, 38.10)	0.290	20.24 (15.16, 22.95)	29.69 (15.92, 39.62)	31.24 (23.19, 37.69)	0.196	Exploratory: N vs HC: 0.028; Other comparisons: NS
Plasmablast%,median (Q1,Q3)	0.06 (0.04, 0.07)	0.25 (0.01, 0.16)	0.134	0.09 (0.05, 0.25)	0.03 (0.01, 0.16)	0.02 (0, 0.03)	0.059	Exploratory: N vs LN: 0.037; Other comparisons: NS
Plasmablast/B cell%, median (Q1,Q3)	1.36 (1.03, 2.4)	1.47 (0.79, 5.85)	0.425	4.37 (1.59, 8.37)	1.47 (0.82, 5.6)	1.39 (0.69, 4.08)	***0.047***	N vs HC: 0.034, N vs LN: 0.007, Other comparisons: NS

P-values for comparisons between HCs and All SLE patients were calculated using the Mann-Whitney U test. Overall P value: P values for comparisons across the four groups were calculated using the Kruskal-Wallis H test for continuous variables and the Chi-square test for sex distribution. Pairwise P values: For variables with a significant Overall P value (P < 0.05), pairwise comparisons were performed using Dunn’s post-hoc test with Bonferroni correction. Results are shown in bold. For variables with a non-significant Overall P value, specific pre-specified pairwise comparisons were performed as exploratory analyses. These P values are indicated with an asterisk (Exploratory) and should be interpreted with caution*HC *Healthy Control, *LN *Lupus Nephritis, *N* Newly diagnosed SLE, *S* Stable SLE

Further analysis of SLE subgroups showed distinct patterns (Table 2). Kruskal–Wallis test revealed significant differences across groups for the proportion of naïve B cells within total B cells (Naïve B/B cell%, P = 0.022) and the proportion of plasmablasts among B cells (plasmablast/B cell%, P = 0.047). Post-hoc pairwise comparisons showed that the Naïve B/B cell% was significantly elevated in newly diagnosed SLE patients compared to both HCs (P = 0.049) and the LN group (P = 0.047). Conversely, the plasmablast/B cell% was significantly expanded in newly diagnosed SLE patients compared to HCs (P = 0.034), and was notably lower in the LN group compared to the newly diagnosed group (P = 0.007).

For variables where the overall Kruskal–Wallis test was not statistically significant (e.g., memory B/B cell%, P = 0.196; plasmablast%, P = 0.059), we performed exploratory pairwise comparisons for specific, pre-specified contrasts of interest. A significant reduction in the relative proportion of memory B cells within the B-cell compartment (memory B/B cell%) was observed in the newly diagnosed SLE group compared to HCs (P = 0.028). Similarly, exploratory analysis in plasmablast frequency (plasmablast%) suggested a higher frequency in newly diagnosed compared to LN patients (P = 0.037). No other pre-specified pairwise comparisons for these variables reached statistical significance.

The most consistent alterations were observed in the absolute frequencies. Both naïve B% and memory B% showed significant overall differences (P = 0.008 and P < 0.001, respectively). Post-hoc tests confirmed that the frequency of naïve B cells was reduced in the stable and LN group versus HCs (P = 0.027 and P = 0.013, respectively), and that memory B% was significantly decreased in all SLE subgroups (newly diagnosed, stable, and LN) compared to HCs (P < 0.001, P < 0.001 and P = 0.008, respectivel.

### Diagnostic value of B-cell subsets for newly diagnosed SLE

**Fig. 3:**
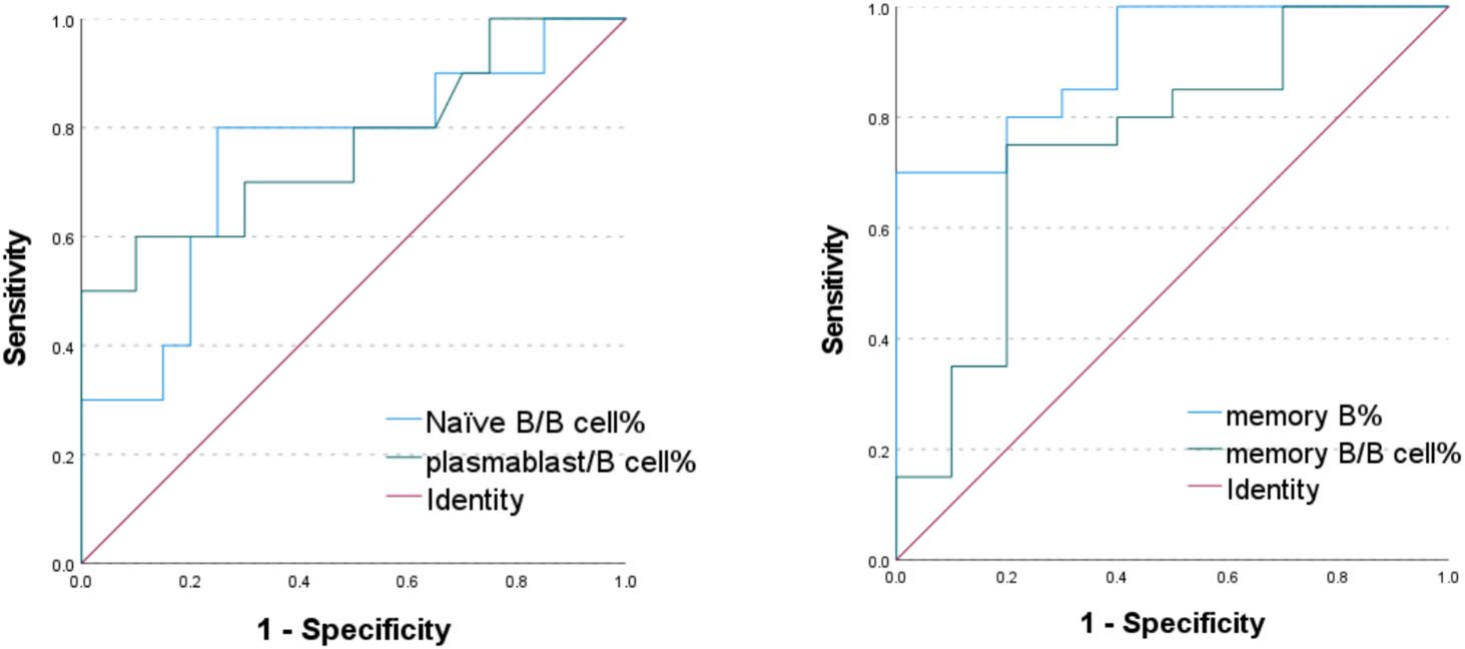
Receiver operating characteristic (ROC) curves of different B-cell subsets for diagnosing SLE

The diagnostic performance of B-cell subsets was evaluated for distinguishing newly diagnosed SLE patients (n = 20) from healthy controls (n = 20) using Receiver Operating Characteristic (ROC) curve analysis. (Table 3). Among the subsets evaluated, the absolute frequency of memory B cells (memory B%) demonstrated outstanding diagnostic performance, achieving the highest Youden’s index (0.60) and an excellent area under the curve (AUC) of 0.905. At the optimal cutoff value of 0.825%, it showed balanced sensitivity and specificity (both 80%), indicating a strong capacity to discriminate SLE patients from healthy controls. The plasmablast-to-B-cell ratio (plasmablast/B cell%) also exhibited good diagnostic utility, with an AUC of 0.768. It displayed high specificity (85%) at a cutoff of 3.235%, suggesting its particular usefulness in ruling out non-SLE individuals (i.e., low false-positive rate), although its sensitivity was moderate (60%). The relative proportion of memory B cells within the total B-cell pool (memory B/B cell%) also showed significant diagnostic value, with an AUC of 0.750. At a cutoff of 23.19%, it maintained high specificity (80%) with reasonable sensitivity (75%). In contrast, the naïve B-cell proportion (Naïve B/B cell%) showed the lowest, though still acceptable, discriminative ability among the tested markers, yielding an AUC of 0.745. At the cutoff of 77.16%, it provided a sensitivity of 70% and a specificity of 75% (Fig. 3).

**Table 3:** Diagnostic efficacy analysis of different B-cell subsets in SLE.

Variables	Sensitivity	Specificity	Youden’s index	Cutoff	AUC
Memory B%	0.80	0.80	0.60	0.825	0.905
Memory B/B cell%	0.75	0.80	0.55	23.19	0.750
Plasmablast/B cell%	0.60	0.85	0.45	3.235	0.768
Naïve B/B cell%	0.70	0.75	0.45	77.16	0.745

### Association between B-cell subsets and ANA titers in SLE patients

SLE patients were stratified into three groups based on their anti-nuclear antibody (ANA) titers: a negative group, a low-positive group (1:100 +), and a high-positive group (1:320 +). The groups were comparable in terms of sex distribution and mean age (p > 0.05), indicating that demographic differences did not confound the subsequent immunological analyses. Analysis of B-cell subsets revealed several significant differences across the ANA titer groups (Table 4): The absolute proportion of naïve B-cells (naïve B%) showed a significant decreasing trend across groups with higher ANA titers (p = 0.027). However, the relative proportion of naïve B-cells within the total B-cell compartment (Naïve B/B cell%) remained unchanged (p = 0.291). The absolute frequency of memory B-cells (memory B%) was profoundly and significantly lower in both ANA-positive groups compared to the ANA-negative group (p < 0.001). In contrast, the relative proportion of memory B-cells (memory B/B cell%) was not significantly different among the groups (p = 0.271). The percentage of plasmablasts among total nucleated cells (plasmablast%) did not differ significantly (p = 0.911). However, the relative proportion of plasmablasts within the total B-cell pool (plasmablast/B cell%) increased significantly with higher ANA titers (p = 0.018). The high-positive ANA group (1:320 +) exhibited the highest median value (3.47%).

**Table 4:** Comparison of B-cell subsets stratified by ANA titer in patients with SLE.

Variables	Negative (n = 16)	1:100 + (n = 28)	1:320 + (n = 20)	p
Sex, n (%)				0.773
Male	3 (19)	3 (11)	1 (5)	
Female	13 (81)	25 (89)	19 (95)	
Age, mean ± SD	51.67 ± 10.54	44.39 ± 12.42	42.15 ± 13.41	0.279
Naïve B%, median (Q1,Q3)	2.36 (1.45, 3.46)	1.22 (0.62, 1.87)	1 (0.42, 2.68)	***0.027***
Naïve B/B cell%, median (Q1,Q3)	68.1 (62.2, 75.88)	73.64 (64.85, 80.9)	74.9 (62.64, 90.09)	0.291
Memory B%, median (Q1,Q3)	1.1 (0.65, 1.35)	0.43 (0.27, 0.76)	0.33 (0.27, 0.68)	** < *0.001***
Memory B/B cell%, median (Q1,Q3)	31.75 (23.4, 37.8)	26.36 (19.03, 34.94)	25.1 (9.91, 35.83)	0.271
Plasmablast%,median (Q1,Q3)	0.05 (0.02, 0.07)	0.03 (0.01, 0.08)	0.03 (0.02, 0.1)	0.911
Plasmablast/B cell%, median (Q1,Q3)	1.22 (0.6, 2.32)	1.47 (0.75, 5.86)	3.47 (1.05, 5.87)	***0.018***

### Association of B-cell subsets with anti-dsDNA and anti-Sm antibody status in SLE patients

SLE patients were categorized into positive and negative groups according to their anti-dsDNA and anti-Sm antibody status (Table 5). Demographic characteristics, including sex distribution and mean age, did not differ significantly between seropositive and seronegative individuals for either antibody (all p > 0.05), suggesting that the immunological differences observed are unlikely to be influenced by these variables. Comparative analysis of B-cell subsets revealed distinct profiles associated with each autoantibody: Among anti-dsDNA positive patients, significant reductions were observed in the absolute levels of naïve B-cells (median: 0.98% vs. 1.66%; p = 0.045), and memory B-cells (median: 0.29% vs. 0.65%; p = 0.003) compared with the anti-dsDNA negative group. In contrast, although the absolute plasmablast count was similar between the two groups (p = 0.807), the proportion of plasmablasts within the total B-cell compartment was markedly higher in anti-dsDNA positive patients (median: 3.36% vs. 1.23%; p = 0.016). In the case of anti-Sm antibody, positivity was not associated with significant changes in the absolute counts of total, naïve, or memory B-cells (all p > 0.05). However, anti-Sm positive patients exhibited a non-significant trend toward an elevated absolute plasmablast count (p = 0.052) and a significantly increased relative plasmablast frequency (median: 4.24% vs. 1.22%; p = 0.006) compared with their anti-Sm negative counterparts.

**Table 5:** Comparison of B-cell Subsets in SLE patients stratified by Anti-dsDNA and Anti-Sm antibody status.

Variables	dsDNA			SM		
	Negative (n = 45)	Positive(n = 19)	P value	Negative (n = 42)	Positive(n = 22)	P value
Sex, n (%)			0.529			0.21
Male	3 (7)	2 (11)		3 (19)	3 (11)	
Female	42 (93)	17 (89)		13 (81)	25 (89)	
Age, mean ± SD	45 ± 11.93	41.22 ± 16.55	0.421	45.71 ± 12.29	39.67 ± 13.55	0.148
Naïve B%, median (Q1,Q3)	1.66 (0.81, 3)	0.98 (0.51, 1.5)	***0.045***	1.65 (0.95, 2.76)	1.39 (0.56, 2.89)	0.696
Naïve B/B cell%, median (Q1,Q3)	69.54 (62.16, 80.77)	74.9 (63.41, 79.42)	0.707	69.74 (57.76, 83.43)	71.76 (63.57, 78.14)	0.901
Memory B%, median (Q1,Q3)	0.65 (0.38, 1.14)	0.29 (0.17, 0.32)	***0.003***	0.66 (0.32, 1.13)	0.6 (0.31, 0.8)	0.439
Memory B/B cell%, Mmedian (Q1,Q3)	29.69 (19.23, 37.69)	25.1 (19.07, 35.7)	0.719	30.2 (16.57, 41.72)	26.58 (20.75, 36.43)	0.799
Plasmablast%,median (Q1,Q3)	0.04 (0.02, 0.08)	0.03 (0.02, 0.09)	0.807	0.04 (0.01, 0.07)	0.07 (0.02, 0.18)	0.052
Plasmablast/B cell%, median (Q1,Q3)	1.23 (0.69, 2.98)	3.36 (1.74, 6.54)	***0.016***	1.22 (0.67, 2.46)	4.24 (1.23, 7.85)	***0.006***

## Discussion

B lymphocytes play a central role in the adaptive immune response in systemic lupus erythematosus (SLE), contributing to the production of autoantibodies, presentation of self-antigens, and activation of reactive T cells^7^. Numerous studies have indicated abnormal expression of B cells in the peripheral blood of patients with autoimmune diseases^8,9^. In these patients, aberrant activation and differentiation of B cells can alter the inflammatory cytokine milieu, dysregulate transcription factor activity, and affect relevant signaling pathways. It has been proposed that the pathological mechanism of SLE is closely linked to the process of B-cell development. According to this view, foreign antigens binding to self-antigens may activate B cells, leading to their proliferation and differentiation into plasma cells, which produce large quantities of autoantibodies, thereby inducing inflammatory damage in various organs and tissues. This suggests that peripheral B cells in SLE patients may undergo altered differentiation states, and such dysregulated B-cell differentiation could play an integral role in the pathogenesis and progression of SLE^10^. Studies have shown that alterations in peripheral B-cell subsets are closely associated with SLE disease activity, which may provide important insights for predicting disease relapse^11^. In this study, by analyzing the distribution characteristics of peripheral blood B-cell subsets in SLE patients with different disease states, we reveal the central role of abnormal B-cell activation in the immunopathogenesis of SLE.

Memory B cells represent a critical component of the adaptive immune system, responsible for long-term antibody-mediated immunity. These cells are generated following initial antigen exposure—such as to pathogens or vaccines and can persist for extended periods. Upon re-encounter with the same or a similar antigen, memory B cells rapidly activate and differentiate into plasma cells, which produce high-affinity antibodies to efficiently neutralize pathogens and prevent their spread and reinfection^12^. We observed a significant reduction in memory B cells across all SLE patient groups, a finding consistent with several recent studies^13^. The persistent loss of memory B cells may be related to impaired clearance of autoreactive B cells or defective peripheral survival^14^. Notably, this reduction was more pronounced in active disease states (such as newly diagnosed SLE and LN), suggesting a close association with disease activity. It is worth noting that an expansion of atypical memory B-cell subsets has been reported in SLE and linked to renal involvement^15^. This implies that the observed decrease in total memory B cells might coincide with the expansion of functionally abnormal subsets, warranting further subpopulation analysis.

Corresponding to the changes in memory B cells, plasmablasts were significantly expanded in newly diagnosed SLE patients, aligning with the well-documented overproduction of autoantibodies in SLE. The expansion of plasmablasts, as precursors to antibody-secreting cells, reflects active humoral immune responses. Recent studies suggest that aberrant activation of B-cell receptor (BCR) signaling pathways in the autoimmune disease can significantly promote B-cell differentiation into plasma cells^16^. This provides a potential molecular mechanism for the observed plasmablast expansion in SLE. The lower plasmablast proportion in the LN group compared to the newly diagnosed group may be related to immunosuppressive treatment or specific disease stages, suggesting that different phases of SLE might involve distinct patterns of B cell activation.

ROC curve analysis indicated that memory B cells demonstrated outstanding diagnostic efficacy (AUC = 0.905) in distinguishing SLE patients from healthy controls, with both sensitivity and specificity reaching 80%. This finding suggests that memory B-cells could serve as a robust laboratory marker for aiding SLE diagnosis. The percentages of plasmablast (AUC = 0.768) and the memory B cell (AUC = 0.750) among total B cells also showed good diagnostic value, with their high specificities (85% and 80%, respectively) being particularly advantageous for ruling out non-SLE individuals. These findings are consistent with the views of Cao Xuetao’s team, who recently emphasized in a review that single-cell analysis of autoreactive B-cells and the identification of pathogenic B-cell subsets are crucial for understanding SLE pathogenesis and developing targeted therapies^17^. Our study further confirms that detailed analysis of peripheral blood B-cell subsets can not only aid in the diagnosis of SLE but may also provide valuable information for assessing disease activity. It is noteworthy that among the subsets analyzed, the frequency of memory B cells among total nucleated cells demonstrated the highest diagnostic value (AUC = 0.905) for distinguishing SLE patients from HCs, outperforming the frequencies of naïve B cells (AUC = 0.745) and plasmablasts (AUC = 0.768). This hierarchy of diagnostic performance suggests that disturbances in the memory and effector B cell compartments may serve as more potent discriminators than alterations in the naïve B cell pool in this cohort. However, it is important to consider that the frequencies of these circulating subsets are not independent; the relative expansion of one population, such as plasmablasts or double-negative B cells, may inherently contribute to the contraction of others within the shared peripheral B cell niche. Therefore, while the AUC values highlight differential diagnostic utility, they do not directly delineate independent cellular mechanisms or the absolute pathogenic contribution of each subset. Our study showed that B cell subset analysis may serve as a valuable tool for the diagnosis and monitoring of SLE. Specifically, the quantitative analysis of memory B-cells may not only aid in diagnosis but also provide information for assessing disease activity and treatment response. For patients clinically suspected of having SLE but with an atypical autoantibody profile, B cell subset analysis could offer supplementary diagnostic evidence.

A key finding of this study is the significant association between different B cell subsets and specific autoantibodies. We observed that as ANA titers increased, the proportion of naïve B cells progressively decreased, while the plasmablast proportion significantly increased. This gradient pattern suggests that the intensity of the autoimmune response is closely linked to the state of B-cell differentiation, with high ANA titers potentially reflecting more active B cell activation and differentiation. Particularly noteworthy is the observation that different specific autoantibodies were associated with distinct patterns of B cell dysregulation, Anti-dsDNA antibody-positive patients exhibited significant B cell differentiation abnormalities, including reduced naïve and memory B cells, and a higher plasmablast proportion. Anti-dsDNA antibody is a characteristic autoantibody in SLE, closely associated with severe clinical manifestations such as renal involvement^18^. Our findings support this notion, indicating that the production of anti-dsDNA antibodies is linked to systemic B cell activation. Anti-Sm antibody-positive patients primarily showed a significant increase in plasmablasts, with no marked changes in other B cell subsets. Anti-Sm antibody is highly specific for SLE and is considered a marker antibody. This relatively specific B cell activation profile suggests that the production of different autoantibodies might involve distinct immunopathological mechanisms and B cell activation pathways. Our study, from the perspective of peripheral B-cell differentiation, further confirms the critical role of aberrant B-cell development and differentiation in SLE pathogenesis. the different B-cell abnormality patterns observed in patients positive for different autoantibodies suggest that SLE may comprise distinct immunological endotypes. This finding aligns with the emerging view of SLE as a highly heterogeneous disease with potentially diverse underlying immunopathogenic mechanisms^19^. Identifying these different immunological endotypes could pave the way for personalized treatment strategies. For example, patients with a significant increase in plasmablasts might benefit more from therapies specifically targeting plasma cells. our results also provide a rationale for B-cell-targeted therapies. Various B-cell-targeting strategies have been extensively studied in SLE in recent years, including B-cell depletion therapies and targeted therapies against B-cell survival factors like BAFF^20^. Understanding the patterns of B cell dysregulation across different disease stages and patient subgroups could help optimize these targeted therapies and improve treatment precision.

Although this study provides valuable insights into the changes in B cell subsets in SLE patients, several limitations should be acknowledged. First, this was a single-center study with a limited sample size, particularly in certain subgroups (e.g., LN patients), which might affect the stability of the results. Second, our analysis is based on the relative frequencies (percentages) of B-cell subsets. While informative of cellular composition, these data do not provide absolute cell counts, limiting our ability to distinguish between true cellular expansion/contraction and proportional shifts within the lymphocyte compartment. Future studies incorporating absolute quantification are warranted. Third, our flow cytometry panel was designed to capture major functional B cell subsets (naïve, memory, plasmablasts) but lacked additional markers (e.g., CD11c, CD21, CD24) that would allow for more granular phenotyping of atypical or transitional subsets reported in SLE. This limits the depth of our subset analysis. Fourth, the promising diagnostic performance of specific B cell subsets was not validated in an independent cohort, which is essential before any clinical application can be considered. Finally, from a translational perspective, while distinguishing SLE from healthy controls is a necessary first step, assessing the diagnostic utility of these B cell subsets against common SLE mimickers (e.g., other systemic autoimmune diseases) would be more clinically relevant and remains an important direction for future research.

## Conclusion

This study systematically analysed the distribution characteristics of peripheral blood B cell subsets in SLE patients and their relationship with disease states and autoantibodies. Our results indicate that SLE patients exhibit significant abnormalities in B cell differentiation, primarily characterized by the reduction of memory B cells and expansion of plasmablasts. These alterations are closely associated with disease activity and specific autoantibodies, particularly anti-dsDNA and anti-Sm antibodies. Memory B cells demonstrated outstanding diagnostic value for SLE, suggesting that B cell subset analysis can be a useful auxiliary diagnostic tool. These findings not only deepen our understanding of the immunopathogenesis of SLE but also provide new insights for the diagnosis, endotyping, and treatment of this complex disease. Future studies should focus on validating the clinical utility of these B cell markers and exploring precise treatment strategies targeting different B cell subsets, ultimately aiming to improve the long-term prognosis for SLE patients.

## Data Availability

The data supporting the findings of this study are available from the corresponding author upon reasonable request.
